# Micronutrient Dietary Intake in Latina Pregnant Adolescents and Its Association with Level of Depression, Stress, and Social Support

**DOI:** 10.3390/nu9111212

**Published:** 2017-11-04

**Authors:** Angelie Singh, Caroline Trumpff, Jeanine Genkinger, Alida Davis, Marisa Spann, Elizabeth Werner, Catherine Monk

**Affiliations:** 1 Division of Behavioral Medicine, Department of Psychiatry, Columbia University Medical Center, New York, NY 10032, USA; angelie@post.bgu.ac.il (A.S.); ad3225@cumc.columbia.edu (A.D.); mns2125@cumc.columbia.edu (M.S.); ew150@cumc.columbia.edu (E.W.); cem31@columbia.edu (C.M.); 2 Medical School for International Health, Faculty of Health Sciences, Ben Gurion University of the Negev, Be’er Sheva 84105, Israel; 3 Department of Epidemiology, Mailman School of Public Health, Columbia University, New York, NY 10032, USA; jg3081@cumc.columbia.edu; 4 Herbert Irving Comprehensive Cancer Center, Columbia University Medical Center, New York, NY 10032, USA; 5 New York State Psychiatric Institute, New York, NY 10032, USA; 6 Department of Obstetrics and Gynecology, Columbia University Medical Center, New York, NY 10032, USA

**Keywords:** adolescent, pregnancy, micronutrients, nutritional status, mood

## Abstract

Adolescent pregnant women are at greater risk for nutritional deficits, stress, and depression than their adult counterparts, and these risk factors for adverse pregnancy outcomes are likely interrelated. This study evaluated the prevalence of nutritional deficits in pregnant teenagers and assessed the associations among micronutrient dietary intake, stress, and depression. One hundred and eight pregnant Latina adolescents completed an Automated Self-Administered 24-hour dietary recall (ASA24) in the 2nd trimester. Stress was measured using the Perceived Stress Scale and the Prenatal Distress Questionnaire. Depressive symptoms were evaluated with the Reynolds Adolescent Depression Scale. Social support satisfaction was measured using the Social Support Questionnaire. More than 50% of pregnant teenagers had an inadequate intake (excluding dietary supplement) of folate, vitamin A, vitamin E, iron, zinc, calcium, magnesium, and phosphorous. Additionally, >20% of participants had an inadequate intake of thiamin, riboflavin, niacin, vitamin B6, vitamin B12, vitamin C, copper, and selenium. Prenatal supplement inclusion improved dietary intake for most micronutrients except for calcium, magnesium, and phosphorous, (>50% below the Estimated Average Requirement (EAR)) and for copper and selenium (>20% below the EAR). Higher depressive symptoms were associated with higher energy, carbohydrates, and fats, and lower magnesium intake. Higher social support satisfaction was positively associated with dietary intake of thiamin, riboflavin, niacin, vitamin B6, folate, vitamin B12, vitamin C, vitamin E, iron, and zinc. The findings suggest that mood and dietary factors are associated and should be considered together for health interventions during adolescent pregnancy for the young woman and her future child.

## 1. Introduction

The spread of industrialized agriculture has allowed for a net increase in calories and macronutrients consumed by the United States population, leading to an overall rise in average body mass; however, these gains are not necessarily associated with adequate micronutrient intake [1]. Teenagers in the U.S. typically have energy-dense diets with poor micronutrient content [2]. Inadequate nutrient intake may be particularly detrimental for pregnant adolescents, who have greater nutritional requirements than their non-pregnant peers and pregnant adults because they must nourish not one but two growing bodies [3,4,5,6]. Previous studies have indicated that U.S. pregnant adolescents often do not meet the Institute of Medicine’s (IOM) recommendations for micronutrient intake [7,8,9,10,11]. These studies have found that the mean intake of iron, zinc, folate, vitamin E, calcium, and magnesium often falls below the IOM’s recommended dietary allowance (RDA). This is a matter of concern because vitamins and minerals are essential for healthy gestation and fetal development, and an insufficient intake may affect pregnancy outcomes [12].

Adequate micronutrient intake is a critical factor for maternal mental health: deficiencies in folate, vitamin B12, calcium, iron, selenium, zinc, and magnesium have been associated with symptoms of depression in both pregnant women and postpartum women [13,14,15,16]. On the other hand, emotional problems, such as negative mood and stress, can alter nutritional decision-making and lead to micronutrient deficiencies [17,18].

To our knowledge, only one study has considered maternal distress in relation to micronutrient intake during pregnancy, yielding positive correlations in adult women between increased stress and iron and zinc deficiencies, and a negative correlation between anxiety and vitamin C deficiency [19].

Pregnant adolescents, who are highly at risk for stress and depression as well as inadequate nutrient intake, are an important population with which to examine the overlap of these two exposures, given that these factors are likely interrelated with respect to adverse birth outcomes and neurocognitive impairments in their offspring [20,21]. In the U.S. today, the fastest growing group with teen pregnancies is Hispanic [22].

Our study objectives were twofold: in pregnant Latina adolescents, assess (1) the average micronutrient intake of adolescent pregnant women as it relates to the U.S. recommendation, with and without taking into account supplement intake in the dietary intake calculation, and (2) the associations among micronutrient intake, stress, depressive symptoms, and social support.

## 2. Materials and Methods

### 2.1. Study Design and Population

Nulliparous pregnant adolescents, aged 14–19 and between 12 and 27 gestational weeks, were recruited through the Departments of Obstetrics and Gynecology at Columbia University Medical Center (CUMC) and Weill Cornell Medical College from June 2009 to March 2011. Further information on the design of this study is available elsewhere [23]. Participants were excluded if they were older than 19 years at the estimated time of conception, currently smoking or using recreational drugs, multiparous, or if they lacked fluency in English. All participants provided written informed consent. The Institutional Review Board of the New York State Psychiatric Institute/CUMC approved all procedures. Of 177 enrolled participants, 114 had completed one 2nd trimester 24-hour food recall; of these participants, 108 self-identified as Latina and were selected for analysis.

### 2.2. Procedures

Participants were scheduled to come to the hospital for two consecutive weekdays during their 2nd trimester to complete demographic and medical questionnaires, psychosocial questionnaires (see Instruments), and one 24-hour food recall.

This report covers the 2nd trimester visit, as this is when most pregnant women are engaged in prenatal care and therefore are available for study recruitment, and when adverse diet-related experiences, such as nausea and vomiting, have largely subsided [24].

All participants reported age, family income, employment, high school status, zip code of current residence, and status of relationship with the baby’s father. Participants also answered questions about health problems, current medications, and previous pregnancies. During the study session, research assistants measured participants’ height and weight using standardized scales. Pre-pregnancy Body Mass Index (BMI) was derived by subtracting self-reported gestational weight gain from current weight, and then dividing by the square root of the measured height.

### 2.3. Measures

#### 2.3.1. General stress

Stress was measured using the Perceived Stress Scale (PSS). This 14-item questionnaire measures the degree to which life situations during the previous month are appraised as challenging one’s ability to cope. Participants responded to items on a five-point Likert scale from 0 (never) to 4 (very often). The questionnaire has a total score ranging from 0 to 40, with higher scores indicating higher perceived stress. The scale has adequate reliability (Cronbach’s alphas of 0.84, 0.85, and 0.86 for three different samples) [25].

#### 2.3.2. Pregnancy-Specific Stress

Pregnancy-specific stress, or “prenatal distress”, was measured using the Prenatal Distress Questionnaire (PDQ), a 12-item questionnaire measuring one’s level of concern or worry over weight gain, preterm delivery, having an unhealthy baby, and physical symptoms [26]. Participants responded using a scale from 0 (not at all) to 4 (extremely) with a total score ranging from 0 to 48, a higher score indicating higher pregnancy-related stress. This measure has good reliability (Cronbach’s alphas of 0.81) [26] and successfully predicts birth outcomes, such as gestational age or preterm birth [27].

#### 2.3.3. Depressive Symptoms

The Reynolds Adolescent Depression Scale (RADS) is a 30-item questionnaire that measures depressive symptoms in adolescents based on symptomology for major depression and dysthymic disorder as specified by the Diagnostic and Statistical Manual of Mental Disorders, third edition (DSM-III). To assess the severity of symptomology, the RADS requires participants to respond to each question on a scale from 1 (almost never) to 4 (most of the time), with a total score ranging from 30 to 120, higher scores indicating greater depressive symptoms. This scale has very good reliability (Cronbach’s alphas of 0.91) and is strongly correlated with other self-report measures of depression, including the Beck Depression Inventory [28].

#### 2.3.4. Social Support

Social support was assessed using the Social Support Questionnaire (SSQ), a 27-item questionnaire assessing quality of social support and participants’ perceived satisfaction with that support. This report focuses on the latter measure, that of perceived satisfaction. For each question, participants rate their satisfaction on a scale from 1 (very dissatisfied) to 6 (very satisfied). To compute the total score, items are summed and then divided by the total number of items (27), providing a total score ranging between 1 and 6 [29]. This measure has very good reliability (Cronbach’s alphas of 0.97).

#### 2.3.5. Dietary Intake

Dietary intake was collected using the Automated Self-Administered 24-hour Dietary Recall (ASA24), a computer software program that prompts the user to list and describe the foods eaten in the last 24 hours. It has a simple interface and has been tested for user-friendliness in participants with at least some secondary education [30]. Nutrient components were determined by linked food codes and nutrient values from the USDA (the United States Department of Agriculture) Food and Nutrient Database for Dietary Studies (FNDDS). The 24-hour food recall provided intake values for thiamin, riboflavin, niacin, vitamin B6, folate, vitamin B12, vitamin C, vitamin A, vitamin E, iron, zinc, calcium, magnesium, phosphorous, copper, selenium, energy, carbohydrate, total fat, and protein.

At the time of this study, supplemental intake had not been incorporated into the ASA24. In our analyses, the micronutrient values of a standard supplement provided by ASA24 were added to the reported dietary intake calculation when prenatal supplement intake was reported by the participant. The micronutrient values of this standard supplement are the following: calcium, 200 mg, iron, 28 mg, zinc, 25 mg, vitamin C, 120 mg, thiamin, 1.8 μg, riboflavin, 1.7 mg, niacin, 20 mg, vitamin B6, 2.6 mg, folate, 800 μg, vitamin B12, 8 mg, vitamin A, 1201 μg, and vitamin E, 30 mg. Given adolescents’ inconsistent reliability with respect to actual intake, both results with and without imputation for prenatal vitamins are presented.

### 2.4. Statistical Analysis

Individual micronutrients were summarized using arithmetic means and categorical divisions by success versus failure to achieve the Estimated Average Requirement (EAR) for pregnant adolescents. The percentage of pregnant adolescents reporting an inadequate dietary intake of recommended micronutrients was determined using the EAR cut-point method [31,32]. For micronutrients for which no EAR had been established, the percentage of inadequate intake was determined using the Adequate Intake reference value (AI), a nutritional standard proposed by the Institute of Medicine (IOM) to use when population data is too limited to generate an EAR [33]. Dietary intake was also categorized as success or failure to meet the Recommended Daily Allowance (RDA) of the IOM for pregnant and non-pregnant adolescents. The RDA is calculated from the EAR and represents an estimate of the average daily intake level sufficient to meet the nutrient requirements for 97–98 percent of healthy individuals.

For psychosocial risk factors, quantitative data were prorated when necessary to generate final measurement outcomes. Spearman correlations were used to assess associations between continuous psychosocial risk factors and continuous nutrient outcomes. A partial correlation procedure was used to control for energy intake. A one-way ANCOVA was used to test the associations between micronutrient status (computed as success versus failure to achieve the EAR for pregnant adolescents) and psychosocial factors while controlling for energy intake. For all analyses, a p-value of less than 5% was deemed significant. All analyses were conducted using SAS 9.3.

## 3. Results

### 3.1. Demographic and Participant Characteristics

The demographic and participant characteristics are summarized in Table 1. Most participants (89%) reported a household income below U.S. $50,000, with 37% reporting a household income below $15,000. A majority (57%) was either in the 12th grade or had only a high school education. All resided in the New York boroughs. The mean pre-pregnancy BMI was 25.7 (SD = 6.4), with 22% categorized as obese (BMI ≥30). Most participants (81%) reported current prenatal vitamin use. The mean scores from standardized questionnaires related to perceived stress, prenatal distress, depressive symptoms, and social support are presented in Table 1. The mean value for perceived stress was 27.8 (SD = 6.06, min–max = 12.0–43.0), the mean value for prenatal-specific stress or “prenatal distress” was 6.1 (SD = 2.9, min–max = 0.0–13.0), the mean value for depressive symptoms was 63.6 (SD = 14.5, min–max = 34.0–110.0), and the mean value for social support satisfaction was 5.5 (SD = 0.7, min–max = 2.9–6.0).

### 3.2. Nutrient Summary and Comparisons with U.S. Requirements

**Figure 1:**
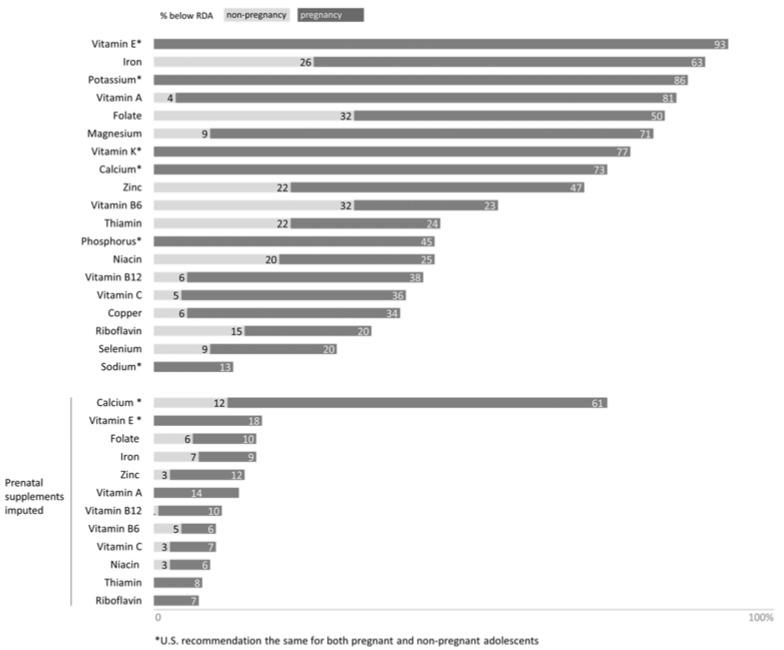
Micronutrient Intake of 108 pregnant Latina adolescents measured with a 24-hour food recall during the 2nd trimester of gestation and compared to the Recommended Dietary Allowance (RDA) for pregnant and non-pregnant adolescents. Figure legend: The bars represent the percentage of participants below the Recommended Dietary Allowance (RDA) for pregnant and non-pregnant adolescents. The proportion in dark grey represents the percentage of participants who do not meet the RDA for adolescent pregnancy (in addition to non-pregnancy), and the proportion in light grey represents the percentage of participants who do not meet the RDA for adolescents only.

The mean energy and nutrient intakes, as well as the proportion of participants below the EAR (nutrient value estimated to meet the requirement of half the healthy individuals in a group), are presented in Table 2. Figure 1 summarizes micronutrient intake—with and without the imputation of prenatal supplement values—in relation to the RDA (nutrient value estimated to meet the requirement of 97–98% of healthy individuals in a group) for the pregnancy and non-pregnancy statuses.

Our sample average energy intake was within the recommended range for the 2nd trimester normal pre-pregnancy BMI of an 18-year-old pregnant female, which is between 2005 and 3173 kcal without accounting for physical activity [34,35].

#### 3.2.1. Micronutrient Status in Pregnant Adolescents Provided by Food Consumption

Without imputing prenatal supplement intake into the calculation of dietary intake, a majority of participants reported an inadequate intake (below the EAR) of folate (72%), vitamin A (69%), vitamin E (90%), iron (81%), zinc (56%), calcium (67%), magnesium (69%), and phosphorous (71%). One out of five participants reported an inadequate intake for all other micronutrients: thiamin (33%), riboflavin (27%), niacin (25%), vitamin B6 (42%), vitamin B12 (33%), vitamin C (36%), copper (25%), and selenium (19%).

When looking at the RDA for pregnant and non-pregnant women (see Figure 1), a large proportion of participants reported nutrients intakes that do not reach not only the recommendation for pregnancy, but also for non-pregnant adolescents. The percentages of participants below the RDA were: 93% for vitamin E, 89% for iron (63% for non-pregnancy), 86% for potassium, 84% for vitamin A (81% for non-pregnancy), 82% for folate (50% for non-pregnancy), 81% for magnesium (71% for non-pregnancy), 77% for vitamin K, 73% for calcium, 69% for zinc (56% for non-pregnancy), 56% for vitamin B6 (23% for non-pregnancy), 46% for thiamin (24% for non-pregnancy), 45% for phosphorous (45% for non-pregnancy), 45% for niacin (25% for non-pregnancy), 44% for vitamin B12 (38% for non-pregnancy), 41% for vitamin C (36% for non-pregnancy), 40% for copper (34% for non-pregnancy), 35% for riboflavin (20% for non-pregnancy), 29% for selenium (20% for non-pregnancy), and 13% for sodium.

In this sample, 10 participants reported an intake below 1000 kilocalories. When we removed this group from analyses, the percentage of inadequate micronutrient intake in *n* = 98 remained the same (without imputing for prenatal supplement). When the data was analyzed for inadequacies in those who reported an energy intake at least 1 SD above the caloric mean (>3138.5 kcal, *n* = 13), these participants had fewer occurrences of inadequate micronutrient intake: thiamin (0%), riboflavin (0%), niacin (0%), vitamin B6 (0%), folate (23%), vitamin B12 (15%), vitamin C (30%), vitamin A (31%), vitamin E (46%), iron (15%), calcium (31%), magnesium (15%), phosphorous (31%), copper (0%), and selenium (0%).

#### 3.2.2. Micronutrient Status in Pregnant Adolescents Provided by Food Consumption and Dietary Supplement Intake

When dietary prenatal supplements were imputed into the calculation, the percentage of inadequate intake calculated from the EAR decreased as follows: folate (15%), vitamin A (12%), vitamin E (20%), iron (17%), zinc (7%), riboflavin (17%), niacin (8%), thiamin (7%), vitamin B6 (7%), vitamin B12 (10%), and vitamin C (11%). The reduction of the percentage of inadequate intake was weaker for calcium, with an improvement from 66% to 61%. When the analysis included only pregnant women reporting prenatal supplement intake, the dietary intake for the above-mentioned micronutrients was above the EAR for every participant except for calcium (57% below EAR) and vitamin A (26% below EAR). Of importance, two thirds of our participants fell below the EAR for magnesium (69%) and phosphorous (71%), and the EAR was not met for one out of five participants for copper (25%) and selenium (19%).

### 3.3. Nutrient Intake and Psychosocial Risk Factors

The correlations between both macro- and micronutrients and psychosocial risk factors are presented in Table 3. A positive association was found between depressive symptoms and energy (*r* = 0.25, *p* < 0.01), carbohydrates (*r* = 0.24, *p* < 0.05), and fat intake (*r* = 0.22, *p* < 0.05). Depressive symptoms were positively associated with vitamin E (*r* = 0.23, *p* < 0.05), iron (*r* = 0.25, *p* < 0.01), and selenium (*r* = 0.23, *p* < 0.05) with the imputation of prenatal supplement intake. These results were not found when controlling for caloric intake. Finally, a negative association was found between magnesium intake and depressive symptoms (*r* = −0.20, *p* < 0.05) when controlling for caloric intake.

Prenatal distress was associated with lower vitamin B6 (*r* = −0.23, *p* < 0.05) and vitamin C (*r* = −0.22, *p* < 0.05) intake, although these associations did not hold when imputing for prenatal supplement intake and while controlling for energy intake.

Social support was positively associated with dietary intake of thiamin (*r* = 0.29, *p* < 0.01), riboflavin (*r* = 0.23, *p* < 0.05), niacin (*r* = 0.24, *p* < 0.05), vitamin B6 (*r* = 0.31, *p* < 0.01), folate (*r* = 0.29, *p* < 0.01), vitamin B12 (*r* = 0.19 , *p* < 0.05), vitamin C (*r* = 0.30 , *p* < 0.01), vitamin E (*r* = 0.22, *p* < 0.05), iron (*r* = 0.26, *p* < 0.01), and zinc (*r* = 0.26, *p* < 0.01) when prenatal supplement intake was imputed and the analysis controlled for caloric intake.

Significant results of the group comparison between micronutrient status (categorized as success versus failure to achieve the EAR for pregnant adolescents) and psychosocial factors (while controlling for energy intake) are presented in Table 4. Mean specific pregnancy-related stress (prenatal distress) was higher for pregnant women having a calcium intake below the EAR (*p* = 0.0442) without imputation for prenatal supplement; this effect was not observed when imputing for prenatal supplement intake. Mean depressive symptoms were higher for pregnant adolescents above the EAR for zinc (*p* = 0.0412) and vitamin E (*p* = 0.0359) than for pregnant adolescents with an intake below the EAR when imputing for prenatal supplement intake. Finally, higher mean social support was found in pregnant adolescents with an intake above the EAR for folate (*p* = 0.0008), iron (*p* = 0.0020), vitamin C (*p* < 0.0001), and riboflavin (*p* = 0.0020).

## 4. Discussion

### 4.1. Micronutrient Status in Pregnant Adolescents Provided by Food Consumption

When limited to food consumption (excluding dietary prenatal supplement intake), our results showed important inadequacies in the micronutrients status of Latina pregnant adolescents, the nation’s fastest growing group of pregnant teenagers. Our findings were similar to national data on non-pregnant adolescents [3]. Our results convey slightly poorer dietary characteristics than those found in non-pregnant female adolescents in a 2012 study using data from the most recent National Health and Nutrition Examination Survey (NHANES), which estimated usual intake by averaging 3-day food records (excluding dietary supplements) in girls aged 14–20 (*n* = 3100). In that study, 99% of the adolescents failed to meet the EAR for potassium, 54% for vitamin A, 98% for vitamin E, 14% for iron, 29% for zinc, 82% for calcium, 86% for magnesium, and 41% for phosphorous [3]. Our study found lower percentages of pregnant adolescents met criteria for adequate intake for vitamin A (69%), iron (87%), zinc (61%), and phosphorous (77%), but slightly higher percentages for vitamin E (90%), calcium (67%), and magnesium (69%). Overall, over 60% of adolescents reported inadequate intakes of either folate, vitamin A, vitamin E, iron, zinc, calcium, magnesium, and/or phosphorous on a randomly chosen day during the second trimester. Importantly, based on the RDAs for pregnant and non-pregnant adolescents, we found that a substantial proportion of our population do not meet the recommendation not only for pregnancy but also for non-pregnant adolescents in general.

Regarding pregnant adolescent populations specifically, our results are comparable to those in a previous study evaluating rates of micronutrient inadequacy [36]. In that study, 96 pregnant adolescents completed a single 24-hour recall in their second trimester, yielding the following similar results (excluding dietary supplements): inadequate intakes of vitamin C (40% versus our 36%), B6 (43% versus our 42%), iron (81% versus our 81%), copper (31% versus our 25%), zinc (51% versus our 56%), vitamin E (94% versus our 90%), calcium (71% versus our 67%), thiamin (21% versus our 33%), and niacin (18% versus our 25%). The earlier study did report slightly better intakes of folate (36% inadequate versus our 72%), vitamin A (52% inadequate versus our 69%), riboflavin (11% inadequate versus our 27%), and B12 (16% inadequate versus our 33%), and a slightly worse intake of magnesium (87% inadequate versus our 69%) [36].

We considered whether low energy intake was a factor in inadequate micronutrient intake by removing the subset of participants who reported a 24-hour below 1000 kilocalorie intake. No differences in percentages of inadequate micronutrient intake were observed.

We also analyzed our data for high energy intake, and did note a decrease in inadequate micronutrient intake in this small subsample of n = 13. Nevertheless, even in this group, between 2 and 6 of the 13 participants reported inadequate intake for folate, B12, vitamin C, vitamin A, vitamin E, iron, calcium, magnesium, and phosphorous. These findings suggest that the consumption of energy may modify micronutrient inadequacy but does not guarantee protection against it.

### 4.2. Micronutrient Status in Pregnant Adolescents Provided by Food Consumption and Prenatal Supplement Intake

U.S. public health institutions have promoted supplementation during pregnancy because of the essential roles played by these vitamins and micronutrients for maternal and fetal health. To assess the relative contribution of supplementation in our population, the value of a standard prenatal vitamin was added to the dietary nutrient sums of those who reported prenatal supplement use (81%). As a result, the nutritional intake of folate, vitamin A, vitamin E, iron, zinc, riboflavin, niacin, thiamin, vitamin B6, vitamin B12, and vitamin C were greatly improved. When analyzed with this approach, pregnant women reporting prenatal supplement intake met the EAR for all of the above-mentioned micronutrients. However, it is important to note that compliance with prenatal supplement recommendations is inconsistent. The USDA Dietary Guidelines for Americans (DGA) recommends the consumption of nutrient-rich foods rather than the intake of dietary supplements to meet nutritional recommendations [37]. Indeed, the long-term effects of some supplements remain unclear [38]. Our results suggest that for pregnant adolescents to meet U.S. standards independently of prenatal vitamin supplementation, their dietary habits would have to fundamentally change to include more nutrient dense foods.

Moreover, a large proportion of our participants was below the EAR for calcium (61%), even after imputing for prenatal supplement intake. In addition, neither magnesium nor phosphorous are included in standard prenatal supplements. Thus, the two-thirds of our population who reported intake below the EAR for these micronutrients would need to alter their dietary behaviors or seek additional supplementation to meet recommendations. This also holds for the one-out-of-five participants who did not meet the EAR for copper and selenium, as none of these micronutrients are included in standard prenatal supplements.

An adequate calcium and magnesium intake assures optimal bone mineralization and cellular metabolism during adolescence. Though little has been reported on the direct effect of inadequate intake on adolescent maternal health and fetal development, inadequate calcium and magnesium appears to increase the risk of specific diseases, such as preeclampsia and intra-uterine growth restriction in adults [39]. An impairment of intake may pose risks not yet identified to the growing adolescent and her developing baby.

### 4.3. Psychosocial Factors and Nutrient Intake

In our sample of teenage adolescents, higher reported depressive symptoms were associated with higher energy, carbohydrate, and fat intake. Previous studies have suggested that unhealthy dietary choice could affect one’s biology, leading to the development of depression. The western and high fat and high sugar dietary patterns have been found to be associated with a higher risk of depression [40,41,42,43,44]; however, some reports did not find this association [45]. The ‘comfort food hypothesis’ suggests that chronic stress can promote a coping strategy leading to higher macronutrient intake and preference towards food containing more carbohydrates and saturated fats. Previous studies have found an association between mild depressive symptoms in pregnancy and unhealthy dietary choice [46,47] and binge eating [48]. Given the cross-sectional nature of this study, our results can be interpreted as consistent with both directions of effects, which indeed may be bi-directional processes.

These findings emphasize the importance of specific interventions for pregnant teenagers to ensure both distress reduction and healthy dietary choices. Evidence indicates that both depression [21] and an unhealthy diet during pregnancy have been associated with adverse developmental outcomes in offspring, such as behavioral problems and emotional dysregulation [47,49].

Depressive symptoms were higher for pregnant adolescents above the EAR for zinc and vitamin E than for pregnant adolescents with intake below the EAR. This may be explained in part by an increased consumption of processed foods, which often contain enriched flours high in zinc and vegetable oils high in vitamin E. Stress and depressive symptoms have been shown to alter food patterns, resulting in an increased selection of less nutritious food, particularly high fat and sugar food and processed foods, and a decreased selection of fruits and vegetables [46,47,50,51,52,53]. Our findings are consistent with other prior research demonstrating positive correlations between energy and fat consumption and depressive symptoms. Thus, rather than participants seeking out specific whole foods naturally high in zinc and vitamin E, it may be that their micronutrient results are better explained by the consumption of processed foods. The mean difference in depression scores observed between participants above (zinc, 64.6 and vitamin E, 64.9) and below (zinc, 54.8 and vitamin E, 57.5) the EAR for zinc and vitamin E is small given the range (30–120) of the scale. The same is true for the difference in prenatal distress score observed for teenagers above (5.1) and below (6.6) the EAR for calcium with a scale ranging from 0 to 48. Therefore, the clinical significance of these findings is difficult to apprehend. To the best of our knowledge, this is the first study showing a negative association between magnesium intake and depressive symptoms in adolescent pregnant women. In previous reports, an association between a low intake of magnesium, anxiety, and depressive symptoms has been observed in preclinical studies [54,55,56], as well as in cross-sectional studies investigating non-pregnant adult populations [40,57,58,59]. A prospective study with men aged 20 years found that a low magnesium intake may have an effect on the risk of developing depression [60]. This may be explained by the fact that biological pathways related to the pathophysiology of depression are modulated by magnesium [61]. Indeed, preclinical studies have found that mice under a magnesium-deficient diet showed dysregulation of the hypothalamic-pituitary-adrenal axis and an increased stress response [55]. Although in need of replication, our results on magnesium are a matter of concern as magnesium is not included in prenatal supplements.

Finally, our findings showed that higher satisfaction with social support was associated with a higher intake of thiamin, riboflavin, niacin, vitamin B6, folate, vitamin B12, vitamin C, vitamin E, iron, and zinc (including dietary prenatal supplements and controlling for overall caloric intake). This is in accordance with a previous report showing that adolescents reporting more social support resources engage in more positive health behaviors [62], including nutritional health behavior [63]. With regard to pregnancy specifically, an association has been reported between poor social support and inadequate weight gain during pregnancy [64,65].

These findings emphasize the potential importance of the social environment in determining healthy nutritional choices.

#### Strengths and Limitations of the Study

Observations from this study are strengthened by the magnitude of effects found within a limited sample size. The homogeneity in demographic characteristics likely improved the detection of differences in diet. Our reliance on one 24-hour food recall may have reduced the internal validity of our findings; nonetheless, a one-time 24-hour recall reduces error compared to the longer interval of recollection for food frequency questionnaires utilized in other studies [32]. Importantly, prenatal supplements were imputed using a standard supplement to estimate their relative contribution to micronutrient intake in our population, and therefore do not reflect the actual intake. It also is important to note that the risk of recall bias is a matter of concern in adolescent populations, particularly in those with high psychosocial risk. Future studies investigating micronutrient intake in pregnant adolescents should report on dietary intake at different time points in pregnancy via a 24-hour food recall that includes prenatal supplements in their micronutrient calculation. To avoid recall bias and add objective measures to the results, this measure should be complemented by peripheral blood assays to obtain precision with respect to the biological availability of nutrients. Our findings on the associations between psychosocial factors and micronutrients intakes are supported by previous studies in other populations [40,41,42,43,44,54,55,56,57,58,59,64,65]. However, given the number of comparisons made, they should be considered as preliminary and in need of confirmation by future studies. Except for our depression measure (RADS), the psychosocial measures used in this study were not validated in a Latina population and this could limit the reliability and validity of our findings.

## 5. Conclusions

In this study of healthy pregnant adolescents engaged in prenatal care, over 60% reported inadequate dietary intake (independent of supplement intake) of either folate, vitamin A, vitamin E, iron, zinc, calcium, magnesium, and/or phosphorous on a randomly chosen day during the second trimester. Overall, deficiencies in micronutrient intake were identified even in the context of adequate calories and ongoing prenatal care. This suggests that, for pregnant teenagers, a healthy diet cannot be guaranteed by sufficient energy intake and that measures of weight gain obtained during prenatal care may be a poor proxy for the assessment of nutrient adequacy. Specific interventions for teenage pregnant women are needed to ensure adequate micronutrients are provided by nutrient-dense food. When we included a prenatal supplement into the dietary recall nutrient calculation, our results showed that supplement use helped to reach adequate intake for some but not all essential micronutrients. A significant proportion of our participants still showed inadequate intake for copper (25%), selenium (19%), calcium (61%), phosphorous (71%), and magnesium (69%). Of importance, our results showed an association between prenatal depression and a lower intake of magnesium. Finally, higher social support was associated with a higher intake of thiamin, riboflavin, niacin, vitamin B6, folate, vitamin B12, vitamin C, vitamin E, iron, and zinc, demonstrating the importance of the social environment for healthy nutritional choices.

## Figures and Tables

**Table 1:** Demographic, health, and psychosocial characteristics of the study population: 108 pregnant Latina adolescents from New York City.

Variable	N	%	Mean	SD	Min	Max
Age (years)			18.0	1.2	14.0	20.0
In relationship with father						
Yes	89	82%				
No	17	16%				
missing	2	2%				
Borough residence						
Manhattan	77	71%				
Bronx/Queens	31	29%				
Overall family income						
0–$15,000	40	37%				
$16,000–$25,000	39	36%				
$26,000–$50,000	17	16%				
$51,000–$100,000	2	2%				
missing	10	9%				
Employed						
Yes	21	20%				
No	86	80%				
missing	1	1%				
Level in school						
8th grade	2	2%				
9th grade	9	8%				
10th grade	12	11%				
11th grade	23	22%				
12th grade	60	57%				
missing	2	2%				
Pre-pregnancy BMI			25.7	6.4	16.6	47.6
missing	1	1%				
Health Problems						
None	72	69%				
Asthma	13	13%				
Other ^a^	19	18%				
missing	4	4%				
Medication use						
Prenatal vitamins	88	81%				
Antibiotic	16	15%				
Pain relief	16	15%				
Asthma and allergy	8	7%				
Other ^b^	15	14%				
Pregnancy History						
Previous Pregnancy	36	34%				
Previous Abortion	20	19%				
Previous Miscarriage	16	15%				
Perceived Stress ^c^			27.8	6.1	12.0	43.0
missing	1	1%				
Prenatal Distress ^d^			6.1	2.9	0.0	13.0
missing	2	2%				
Depressive Symptoms ^e^			63.6	14.5	34.0	110.0
missing	3	3%				
Social Support ^f^			5.5	0.7	2.9	6.0
missing	2	2%				

^a^ four anemia, three migraine, one lupus, one hypothyroidism, one Type 2 Herpes. ^b^ one synthroid, one antifungal, three antiviral, three antinausea, one additional iron supplement, five laxatives, one histamine receptor 2-antagonist. ^c^ Perceived stress was measured by the Perceived Stress Scale, a 14-item self-report questionnaire that produces a maximum score of 56. Higher scores represent a more stressful experience. ^d^ Prenatal distress was measured by the Prenatal Distress Questionnaire, a 12-item self-reported questionnaire with a maximum score of 48. Higher scores represent greater distress. ^e^ Depressive symptoms were measure by the Reynolds Adolescent Depression Scale, a 30-item questionnaire that results in a maximum score of 120. Higher scores represent greater depression symptomology. ^f^ Social support was measured by the Social Support Questionnaire, a 27-item questionnaire on which responses to all items are averaged to result in a satisfaction score with a maximum score of 6. Higher scores represent a more positive perception of social support. BMI: body mass index.

**Table 2:** Nutritional characteristics of 108 pregnant Latina adolescents measured with a 24-hour food recall during the 2nd trimester of gestation and comparison with the Estimated Average Requirement (EAR).

Nutrient	EAR *^b^*	Mean	SD	Min–Max	Participant Below EAR for Pregnant Adolescents	
					N	%
**Energy (kcal)**	ND	2133.0	1005.8	656.6–5831.6		
**Carbohydrate (g)**	135	288.1 ***	132.4	44.1–782.1	11	10%
**Protein (g/(g/kg))**	0.88	76.7/1.2 ***	41.6/0.7	7.7/0.1–257.0/4.2	42	39%
**Fat (g)**	ND	78.3	48.9	9.7–293.2		
**Thiamin (µg)**	1.2	1.6 *** *^d^*	0.9	0.1–5.3	36	33%
***Thiamin (µg)***	*1.2*	3.1**** ^d^	*1.2*	*0.5–7.1*	*8*	*7%*
**Riboflavin (mg)**	1.2	1.8 *** *^d^*	1.0	0.2–6.3	29	27%
***Riboflavin (mg) ^a^***	*1.2*	3.2 **** ^d^	*1.2*	*0.5–6.7*	*17*	*16%*
**Niacin (mg)**	14	21.0 ***	10.2	1.0–60.3	27	25%
***Niacin (mg) ^a^***	*14*	37.29 **** ^d^	*13.4*	*5.8–80.3*	*8*	*7%*
**Vitamin B6 (mg)**	1.6	1.9 **	0.9	0.2–5.4	45	42%
***Vitamin B6 (mg) ^a^***	*1.6*	4.0 **** ^d^	*1.4*	*0.4–8.0*	*7*	*6%*
**Folate (µg)**	520	434.6 ***	239.4	81.5–1566.6	78	72%
***Folate (µg) ^a^***	*520*	1086.5 **** ^d^	*401.5*	*151.8–2366.6*	*15*	*14%*
**Vitamin B12 (mg)**	2.2	4.1 ***	3.3	0.2–15.5	36	33%
***Vitamin B12 (mg) ^a^***	*2.2*	10.6 **** ^d^	*4.6*	*0.2–23.5*	*10*	*9%*
**Vitamin C (mg)**	66/70	147.3 ***	156.3	0.2–1040.6	39	36%
***Vitamin C (mg)***	*66/70*	245.0 **** ^d^	*164.9*	*10.5–1160.6*	*11*	*10%*
**Vitamin A (µg)**	530/550	543.5 **	632.0	51.0–5356.9	74	69%
***Vitamin A (*µ*g) ^a^***	*530/550*	1522.1 **** ^d^	*692.1*	*51.0–5356.91*	*12*	*11%*
**Vitamin E (mg)**	12	6.8 ***	4.27	0.75–22.7	97	90%
***Vitamin E (mg)***	*12*	31.2 ****	*12.86*	*0.75–52.7*	*20*	*19%*
**Iron (mg)**	23/22	16.0 ***	9.28	1.58–47.8	87	81%
***Iron (mg) ^a^***	*23/22*	38.8 **** ^d^	*14.15*	*5.27–75.8*	*17*	*16%*
**Zinc (mg)**	10.5/9.5	10.5	6.98	1.47–51.0	61	56%
***Zinc (mg) ^a^***	*10.5/9.5*	30.9 **** ^d^	*12.95*	*2.43–76.0*	*7*	*13%*
**Calcium (mg)**	1000/800	854.5 *	541.5	99.6–2483.1	72	67%
***Calcium (mg) ^a^***	*1000/800*	1017.5 **** ^d^	*554.9*	*99.6–2683.1*	*66*	*61%*
**Magnesium (mg)**	335/290	282.1 **	146.1	75.4–914.6	74	69%
**Phosphorus (mg)**	1055/580	1164.2 ****	591.7	196.9–3097.5	77	71%
**Copper (mg)**	0.79/0.80	1.3 ****	0.7	0.2–4.3	27	25%
**Selenium (µg)**	49	94.5	60.2	5.5–427.3	21	19%
**Potassium (mg)**	4700 *^c^*	2928.7	1528.0	288.0–8934.1		
**Sodium (mg)**	1500 *^c^*	3194.0	1660.0	891–8672.0		

*p*-value for one-sample t-test of difference between study sample and the EAR, * *p* < 0.05, ** *p* < 0.01, *** *p* < 0.001, **** *p* < 0.0001. Separate tests were performed for each age group when the EAR values differed (N = 62 for ages 14–18 and N = 46 for ages 19–20). *^a^* Values were corrected by adding the standard value of prenatal supplement when prenatal supplement intake was reported. *^b^* EAR, Estimated Average Requirement based on the Institute of Medicine’s (IOM) recommendations. Where values differed based on age, the EAR for pregnant adolescents ages 14–18 are reported first, followed by the EAR values for ages 19 and above. ND indicates not-yet-determined. *^c^* Adequate Intake is reported where no EAR is yet established by the IOM. *^d^* Mean intake is significantly higher than EAR with supplementation imputed.

**Table 3:** Association between nutritional intake, stress, depressive symptoms, and social support in 108 pregnant Latina adolescents.

	Energy	Carbohydrates	Fats	Proteins	Thiamin	Thiamin ^a^	Riboflavin	Riboflavin ^a^
**Unadjusted coefficients**								
Perceived Stress	0.15	0.15	0.12	0.07	0.02	−0.04	−0.03	0.04
Prenatal Distress	−0.10	−0.09	−0.11	−0.11	−0.19	−0.11	−0.14	−0.09
Depressive Symptoms	0.25 **	0.24 *	0.22 *	0.14	0.11	0.17	0.12	0.19
Social Support	0.01	0.03	0.00	−0.02	0.17	0.29**	0.11	0.23 *
**Energy adjusted coefficients**								
Perceived Stress		0.03	0.02	−0.13	−0.15	−0.12	−0.09	−0.07
Prenatal Distress		0.01	−0.01	−0.04	−0.15	−0.03	−0.10	−0.03
Depressive Symptoms		−0.02	0.06	−0.10	−0.12	0.04	−0.07	0.03
Social Support		0.05	−0.03	−0.05	0.26	0.19 *	0.15	0.23 *
	**Niacin**	**Niacin ^a^**	**Vitamin B6**	**Vitamin B6 ^a^**	**Folate**	**Folate ^a^**	**Vitamin B12**	**Vitamin B12 ^a^**
**Unadjusted coefficients**								
Perceived Stress	0.04	0.08	0.01	0.04	0.00	0.04	−0.13	−0.04
Prenatal Distress	−0.10	−0.05	−0.23 *	−0.14	−0.15	−0.09	−0.14	−0.07
Depressive Symptoms	0.17	0.24 *	0.12	0.21 *	0.07	0.17	0.04	0.15
Social Support	0.09	0.22 *	0.09	0.29 **	0.07	0.29 **	0.03	0.21 *
**Energy adjusted coefficients**								
Perceived Stress	−0.12	−0.03	−0.07	−0.06	−0.11	−0.04	−0.09	−0.12
Prenatal Distress	0.00	0.03	−0.20	−0.1	−0.07	−0.04	−0.05	−0.03
Depressive Symptoms	0.01	0.09	−0.06	0.06	−0.13	0.04	−0.06	0.04
Social Support	0.15	0.24 *	0.13	0.31 **	0.09	0.29 **	0.03	0.19 *
	**Vitamin C**	**Vitamin C ^a^**	**Vitamin A**	**Vitamin A ^a^**	**Vitamin E**	**Vitamin E ^a^**	**Iron**	**Iron ^a^**
**Unadjusted coefficients**								
Perceived Stress	−0.03	−0.02	0.01	0.03	0.21 *	0.17	0.00	0.03
Prenatal Distress	−0.22 *	−0.22 *	−0.13	−0.15	−0.09	−0.06	−0.11	−0.05
Depressive Symptoms	−0.11	−0.05	0.05	0.11	0.18	0.23 *	0.11	0.21 *
Social Support	0.16	0.31 *	0.05	0.12	−0.02	0.23 *	0.06	0.25 **
**Energy adjusted coefficients**								
Perceived Stress	−0.01	−0.08	0.09	−0.06	0.14	0.10	−0.17	−0.08
Prenatal Distress	−0.22 *	−0.19	−0.13	−0.11	0.01	0.00	−0.03	0.02
Depressive Symptoms	−0.16	−0.15	−0.04	−0.03	0.05	0.09	−0.12	0.06
Social Support	0.16	0.30 **	0.05	0.09	−0.04	0.22 *	0.08	0.26 **
	**Zinc**	**Zinc ^a^**	**Calcium**	***Calcium ^a^***	**Magnesium**	**Phosphorus**	**Copper**	**Selenium**
**Unadjusted coefficients**								
Perceived Stress	−0.01	0.00	0.02	0.03	0.06	0.09	0.15	0.14
Prenatal Distress	−0.11	−0.07	−0.14	−0.15	−0.18	−0.12	−0.12	−0.01
Depressive Symptoms	0.09	0.15	0.16	0.11	0.12	0.17	0.19	0.23 *
Social Support	−0.07	0.25 **	0.07	0.12	0.04	0.02	0.05	0.01
**Energy adjusted coefficients**								
Perceived Stress	−0.19	−0.12	−0.03	−0.06	−0.16	−0.02	−0.07	−0.03
Prenatal Distress	−0.02	−0.01	−0.09	−0.11	−0.17	−0.11	−0.08	0.09
Depressive Symptoms	−0.17	−0.01	0.03	−0.03	−0.20 *	−0.07	−0.09	−0.02
Social Support	−0.11	0.26 **	0.08	0.09	0.05	0.02	0.06	0.00

***^a^*** Values were corrected by adding the standard value of prenatal supplement when prenatal supplement intake was reported. *p*-value from Spearman correlation * *p* <0.05, ** *p* <0.01.

**Table 4:** Difference in prenatal distress, depressive symptoms, and social support between adequate and inadequate micronutrient intake.

	Above EAR			Below EAR				
	N	Mean	SD	N	Mean	SD	F	*p*-Value
**Prenatal Distress**								
Calcium	35	5.1	2.5	71	6.6	2.9	3.2	0.0442
**Depressive Symptoms**								
Zinc ^a^	94	64.6	14.5	11	54.8	11.0	3.3	0.0412
Vitamin E ^a^	87	64.9	14.4	18	57.5	13.8	3.4	0.0359
**Social Support**								
Folate ^a^	92	5.4’	0.9	14	4.2	1.9	7.6	0.0008
Iron ^a^	90	5.4	0.9	16	4.3	1.8	6.6	0.0020
Vitamin C ^a^	96	5.4	0.9	10	3.8	2.1	11.0	<0.0001
Riboflavin ^a^	90	5.4	0.9	16	4.3	1.8	6.6	0.0020

**^a^** Values were corrected by adding the standard value of prenatal supplement when prenatal supplement intake was reported. *p*-value from analysis of covariance (ANCOVA); the analysis was adjusted for energy intake. Only significant results are presented. Prenatal distress was measured using the Pregnancy Distress Questionnaire (continuous). Depressive symptoms were measured using the Reynolds Adolescent Depressive Scale (continuous). Inadequate micronutrient intake was measured using the Estimated Average Requirement (EAR) cut-point method (dichotomous).
